# Virtual reality-based intervention in forensic mental health services: a feasibility study of implementation, acceptability, and clinical outcomes

**DOI:** 10.3389/fpsyg.2026.1940486

**Published:** 2026-10-23

**Authors:** Gemma Escuder-Romeva, Ellen Vorstenbosch, Aitor-Eneko Olivé-Albitzur, Verónica Tre-Ortega, Raquel Sanz-Heras, Eduardo Luna-Rodríguez, Mónica Civit-Ramírez, María-Cristina Brophy-Palma, Ezequiel-Jesús Pérez-Sánchez, Noemí Del Prado-Sánchez, María Ribas-Siñol

**Affiliations:** 1 Etiopathogenesis and Treatment of Severe Mental Disorders (MERITT), Sant Joan de Déu Research Institute, Sant Joan de Déu Foundation, Barcelona, Spain; 2 Parc Sanitari Sant Joan de Déu, Barcelona, Spain; 3 Centre for Biomedical Research on Mental Health (CIBERSAM), Madrid, Spain; 4 Swiss Paraplegic Research, Nottwil, Switzerland; 5 Department of Medicine and Translational Research, University of Barcelona, Barcelona, Spain

**Keywords:** emotion regulation, feasibility study, forensic mental health, juvenile justice, rehabilitation, relaxation, virtual reality intervention

## Abstract

**Objective:**

This study evaluated the feasibility, acceptability, and preliminary clinical outcomes of an immersive virtual reality (VR)-based relaxation program implemented as part of routine care in adult and juvenile forensic mental healthcare settings.

**Methods:**

A feasibility study was conducted in two forensic mental healthcare services: an adult prison-based rehabilitation unit (UHRPI; *n* = 21) and a juvenile justice treatment unit (UTJJ; *n* = 8). The 12-week study comprised a four-week pre-intervention phase, a four-week intervention phase including eight VR sessions, and a four-week post-intervention phase. Feasibility, comprising service-user acceptability and professional usability, was the primary outcome. Secondary exploratory outcomes included physiological measures (blood pressure and heart rate), state anxiety (STAI-State), and subjective emotional states assessed immediately before and after each VR session using visual analog scales (VAS). Participant-level behavioral indicators extracted from institutional clinical records were summarized descriptively. Linear mixed-effects models were used to analyze repeated physiological and VAS measures, whereas state anxiety was analyzed using paired-samples *t*-tests.

**Results:**

Acceptability and usability were high in both settings, and the intervention was generally well tolerated. In the UHRPI, significant reductions were observed in systolic blood pressure, heart rate, and state anxiety; no significant physiological or anxiety changes were observed in the UTJJ. In both settings, stress and anxiety decreased and relaxation and perceived connection with nature increased immediately following VR sessions, whereas changes in happiness and craving reached significance in only one setting. Participant-level behavioral indicators showed descriptive reductions in several indicators across study phases, with patterns differing between settings.

**Conclusion:**

The VR-based relaxation program was feasible, acceptable, and well integrated into routine forensic mental healthcare. The intervention was associated with improvements in immediate emotional responses across both settings and with favorable physiological changes in adults. Descriptive behavioral findings suggest potential benefits that warrant further investigation. Larger controlled studies with predefined clinical and safety outcomes are needed to determine effectiveness, establish causal effects, and evaluate long-term implementation.

## 1 Introduction

Forensic mental healthcare lies at the complex intersection of mental health care and criminal justice, providing treatment to individuals with serious mental illness whose behavior has led, or could lead, to offending, with the dual aims of improving mental health and reducing recidivism to enable safe reintegration into society (Mullen, 2000; Arboleda-Flórez, 2006). Service users in these settings frequently present with chronic psychopathology, high levels of emotional dysregulation, cognitive impairments, and elevated risks of institutional violence and recidivism, which makes the delivery of effective care particularly challenging. Many show low treatment motivation, limited literacy skills, and difficulties with abstract reasoning, all of which can reduce engagement in conventional psychotherapeutic approaches (Drieschner et al., 2004; Svensson et al., 2016; Kip and Bouman, 2021).

Additionally, incarceration itself constitutes a major environmental stressor. Individuals must adapt to contexts characterized by rigid routines, reduced autonomy, institutional dependence, and uncertainty regarding legal processes and future prospects (Haney, 2001; Porter and DeMarco, 2019; Stephenson et al., 2021). Emotional suffering during detention is further influenced by cumulative adversity and trauma histories, which increase vulnerability to anxiety, depression, and suicidal behavior, particularly during early phases of imprisonment or following judicial or interpersonal setbacks (Mazher and Arai, 2025; Schnyder et al., 2025). These contextual pressures imply that effective treatment strategies must address not only underlying psychological mechanisms but also the environmental constraints inherent to forensic mental healthcare.

From an organizational perspective, interventions that can be embedded into routine workflows while simultaneously enhancing service users’ well-being, staff safety, and the therapeutic climate are of particular interest, as elevated stress levels in forensic environments are associated with symptom exacerbation, aggression, staff burden, and financial costs (Jalil et al., 2017; Kramarz et al., 2023). Approaches that can rapidly attenuate arousal may therefore have systemic as well as individual benefits. Traditional interventions, especially standard cognitive behavioral therapy, largely rely on verbal reflection explained through dialog, imagination, and insight. These requirements may exceed the cognitive capacities of a considerable proportion of forensic service users and thus, limit treatment responsivity and real-world impact (Kip et al., 2018; Smeijers et al., 2018). Consequently, the field has increasingly advocated for innovative methods that translate therapeutic principles into more concrete, experiential learning formats. At the same time, individuals with severe mental illness may struggle to engage in conventional stress-management techniques such as mindfulness or guided imagery because impairments in attention, memory, and executive functioning can hinder their ability to generate and sustain internal representations of calming scenarios (Millan et al., 2012).

In this context, immersive Virtual Reality (VR) has emerged as a promising technology that can shift clinical work from thinking and talking toward doing and experiencing (Kip and Bouman, 2021). VR systems generate a compelling sense of presence, defined as the subjective illusion of “being there” within a computer-generated environment (Slater, 2018; Sygel and Wallinius, 2021). Through this experiential quality, service users can practice coping strategies, emotion regulation skills, and alternative behaviors in situations that approximate real life while remaining safe and controllable. Moreover, such environments enable clinicians to expose service users to emotionally evocative stimuli without risking harm to others, an essential requirement in forensic care (Mertens and Van Gelder, 2025; Sappelli et al., 2025).

A central treatment priority in forensic mental healthcare is the management of emotional tension and physiological arousal, both of which are known precursors of aggression and disruptive behavior (de Looff et al., 2019; Smeijers et al., 2021). Interventions capable of rapidly reducing autonomic activation may therefore contribute not only to individual well-being but also to institutional safety. Immersive relaxation approaches delivered through VR have shown encouraging results. Virtual calm rooms, for example, provide access to soothing natural environments and have been associated with improved affect and reduced distress among inpatients receiving mental healthcare (Ilioudi et al., 2023, 2025).

Beyond symptom reduction, VR may also compensate for the scarcity of restorative experiences in custodial settings. Contact with nature has repeatedly been associated with emotional recovery, improved mood, and reductions in stress-related outcomes (Hartig et al., 2014; White et al., 2019). When real exposure is impossible, simulated natural environments can elicit comparable short-term benefits (Browning et al., 2020). This compensatory function may be particularly relevant in prisons and secure units, where opportunities for sensory relief are structurally constrained. VR relaxation may also offer concrete opportunities to rehearse and strengthen learned skills in ways that facilitate their transfer to everyday life on the unit, through a temporary sense of being elsewhere (Mertens and Van Gelder, 2025). For populations characterized by low motivation and high avoidance, these experiential properties may be especially valuable.

Despite rapid technological development, the routine implementation of VR in forensic mental healthcare remains at an early stage (Kouijzer et al., 2024; Terkildsen et al., 2024). Practical barriers include equipment costs, technical instability, workflow integration, and the need for staff training (Kip et al., 2018; Andersson et al., 2026), as well as emerging ethical challenges, particularly in youth forensic populations, for example concerning informed consent, data protection, equity, and the persuasiveness of digital tools (Kip et al., 2025). Against this backdrop, feasibility research is crucial, and studies must evaluate not only clinical outcomes but also usability, acceptance among both service users and professionals, and compatibility with everyday service delivery (Hendriks et al., 2023; Woicik et al., 2023).

The present study was designed to address this gap. We implemented an immersive VR relaxation program within routine care in two forensic mental healthcare settings: an adult prison-based rehabilitation unit and a juvenile justice treatment unit. The primary objective was to evaluate the feasibility, acceptability, and usability of integrating VR into routine clinical practice from the perspectives of both service users and healthcare professionals. As secondary exploratory objectives, we examined immediate pre-post changes in subjective emotional state and physiological stress responses across VR sessions, as well as descriptive changes in behavioral incidents, restrictive measures and request for additional medication among participants over the study period. By integrating implementation, experiential, clinical, and organizational perspectives, this study aims to provide preliminary evidence to inform the design of future controlled studies evaluating the effectiveness of immersive VR interventions in forensic mental healthcare.

## 2 Materials and methods

### 2.1 Study design and program context

We conducted a prospective, single-arm feasibility study in two forensic mental health settings, combining a post-intervention assessment of acceptability and usability, with a pre-post evaluation of preliminary clinical and exploratory behavioral indicators of an immersive VR-based relaxation program implemented within routine care. This design was considered particularly appropriate for early-stage program evaluation in complex forensic mental health environments, where controlled study designs are difficult to implement, and real-world feasibility is a primary concern.

The intervention was developed and implemented as part of the Virtual-Scape Project, an innovation program aimed at supporting emotional regulation, and enhancing the therapeutic climate in forensic mental healthcare.

### 2.2 Setting and participants

The study was conducted in two forensic mental health services: the Sant Joan de Déu Intensive Psychiatric Hospitalisation and Rehabilitation Unit (Unidad de Hospitalización y Rehabilitación Psiquiátrica Intensiva, UHRPI), located within the Brians 2 Penitentiary Centre, and the Sant Joan de Déu juvenile justice treatment unit (Unidad de Tratamiento de Justicia Juvenil, UTJJ), located within the Til·lers Educational Centre, a closed correctional facility for adolescents and young adults.

At the time of the study, the UHRPI housed 65 male inpatients aged 18 years or older, while the UTJJ accommodated 20 male and female inpatients aged 14–21 years.

Potential participants were initially identified by the clinical professionals of each unit, who reviewed patients under their care against the predefined inclusion and exclusion criteria. Patients considered potentially eligible on the basis of these criteria were referred to the research team, which conducted the study enrolment and informed consent procedures. Service users were eligible if (a) they were able to provide informed consent and understand the study procedures, and (b) had sufficient proficiency in Spanish or Catalan to follow verbal instructions and complete brief self-report measures. Service users were excluded in case of (a) severe visual impairment or neurological conditions interfering with safe VR use (e.g., epilepsy), (b) acute intoxication or withdrawal, or (c) other clinical or security concerns deemed to make participation unsafe. All eligible participants were invited to participate; 29 consented and were enrolled, none withdrew, and all 29 were included in the analyses. The numbers of patients initially screened, considered eligible, invited, or declining participation were not systematically recorded.

The final sample consisted of 29 participants; 21 service users were recruited from the UHRPI and 8 from the UTJJ, corresponding to approximately 32 and 40% of the bed capacity of the respective units at the time of the study. Because admissions and discharges were not recorded, these figures are approximations relative to unit capacity rather than participation rates among all service users present during the study period. Mean age was 35.1 years (SD = 10.2) in the UHRPI and 16.8 years (SD = 1.03) in the UTJJ. All UHRPI participants were men, as this is a male-only unit; the UTJJ sample comprised six men (75%) and two women (25%). Educational attainment differed between the units: in the UHRPI, 43% of participants (*n* = 9) had completed secondary education, 38% (*n* = 8) had completed only primary education, and the remaining 19% (*n* = 4) held vocational or higher-level qualifications, whereas in the UTJJ all participants had completed secondary education (*n* = 8).

In the UHRPI, the most frequent primary diagnosis was paranoid schizophrenia, present in 11 of 21 cases (52.4%), followed by other psychotic disorders (*n* = 2, 9.5%). The remaining diagnoses were each observed in a single case (4.8% each) and included impulse control and conduct disorder, eating disorder, conduct disorder, bipolar disorder, substance use disorder, schizoaffective disorder, autism spectrum disorder, and chronic delusional disorder.

Comorbid conditions were common, particularly substance use disorders (*n* = 8, 38.1%) and personality disorders (*n* = 6, 28.6%). In addition, four individuals (19%) had a diagnosis of mild intellectual disability.

Participants from the UTJJ presented a heterogeneous set of primary diagnoses, with each condition represented in a single case. Diagnoses included substance use disorder, autism spectrum disorder, adjustment disorder, conduct disorder, depression with anxiety, anxiety disorder, post-traumatic stress disorder (PTSD), and Asperger’s syndrome. Comorbidity was identified in 75% of participants in this group (*n* = 6), and in all cases, the comorbid diagnosis involved a substance use disorder.

### 2.3 Intervention program: the virtual-scape project

The Virtual-Scape intervention was delivered over a 12-week period per participant and comprised three sequential phases: a four-week pre-intervention observation phase (pre), a four-week active intervention phase, and a four-week post-intervention follow-up phase (post). No VR sessions were delivered during the pre- or post-phases.

During the intervention phase, participants received eight immersive VR sessions (Table 1), delivered twice weekly as part of routine care activities. Sessions were delivered individually in a quiet room on the ward using a commercially available standalone VR system (PICO VR all-in-one headset, model A7Q10, Qingdao Chuangjian Weilai Technology Co., Ltd.) running the Virtual Realms (Scape) relaxation program, which provides guided breathing exercises and nature-based immersive environments. Each session lasted between 10 and 16 min and focused on relaxation, breath regulation, and guided meditation within immersive natural virtual environments.

**Table 1:** Session objectives and content.

Week	Session 1		Session 2	
	Objective	Content	Objective	Content
1	Breathing control	Ocean Breeze	Relaxation – Pain relief	Swimming with Dolphins
2	Pain and conscious breathing	Crystal Serenity	Self-connection	Essence – Guided Meditation
3	Attention, connection	Connect	Free relaxation	Lanzarote
4	Virtual sedation	Day Dream	Free relaxation	Alps

The VR sessions were administered by a team of nine professionals. In the UHRPI, six staff members participated, including mental health nurses (*n* = 4; 67%) and mental health nursing assistants (*n* = 2; 33%). In the UTJJ, the intervention was delivered by one mental health nurse (33%) and two clinical psychologists (67%). Different professionals could administer sessions to the same participant depending on routine staffing schedules. All professionals received specific training on the administration of VR-based relaxation to ensure standardized delivery of the intervention across both settings. All study procedures were integrated into standard care pathways to minimize disruption to usual service delivery.

### 2.4 Outcome measures

Feasibility, comprising service-user acceptability and professional usability, was the primary outcome. Physiological measures, state anxiety, and subjective emotional states were secondary outcomes and were examined on an exploratory basis; behavioral indicators were descriptive only. Outcomes were assessed at different time points throughout the study, as described below.

Feasibility (acceptability and usability): Acceptability was assessed once at the end of the four-week intervention phase with a 10-point visual analog scale (VAS; 0 = “none,” 10 = “very much”), on which service users rated satisfaction, comfort, willingness to continue using VR and perceived usefulness. Usability was measured with an *ad hoc* questionnaire based on the System Usability Scale (SUS) (Brooke, 1996). At the end of each participant’s four-week intervention phase, a professional involved in delivering the VR intervention completed the questionnaire using a 5-point Likert scale (1 = “strongly disagree,” 5 = “strongly agree”). Professionals rated willingness to use the device, ease of use, learnability, need for assistance, comfort, perceived safety, and applicability in routine clinical practice, and could provide free-text comments on future implementation. The item on need for assistance was worded in the opposite direction to the other six, so that higher scores indicate a greater perceived need for support; it was not reverse-coded. As one usability assessment was completed for each participant, a total of 29 usability assessments were obtained. Physiological and anxiety outcomes: Physiological indicators of stress, including blood pressure (diastolic and systolic) and heart rate were measured immediately before and after each VR session during the intervention phase. Anxiety was assessed using the State scale of the State–Trait Anxiety Inventory STAI (Spielberger et al., 1983), administered at baseline and post-intervention. Raw scores were converted to percentile scores based on the normative data of the Spanish adaptation, and percentile scores were used in all analyses.

Behavioral indicators: Behavioral indicators were obtained from participants’ institutional clinical records using a standardized data extraction form developed for the Virtual-Scape project. For each participant, clinical staff recorded the frequency of predefined behavioral events occurring during three consecutive four-week periods (pre-intervention, intervention, and post-intervention). All participants remained in their unit for the full duration of the study; none was discharged, transferred or withdrawn. Each participant therefore contributed four consecutive weeks of observation to each of the three phases, giving a constant observation period across phases, participants and settings (84 participant-weeks per phase in the UHRPI and 32 participant-weeks per phase in the UTJJ). Events were documented in the individual clinical record at each shift as part of routine clinical practice; no study-specific observation or recording procedure was introduced, so the documentation procedure was identical across the three phases. The extracted indicators included requests for help due to anxiety, requests for additional medication, violent incidents toward peers, violent incidents toward staff, violence involving center property, behavioral incidents requiring professional intervention, and use of restrictive measures (separation from environment, observation, and mechanical restraint), as well as recorded episodes of substance use.

Subjective appraisals: Subjective emotional states were assessed immediately before and directly after each VR session using visual analog scales (VAS; range 0–10). Service users rated their current levels of stress, relaxation, anxiety, sadness, happiness, perceived connection with nature and craving, with higher scores indicating stronger endorsement of the respective state.

In addition to these predefined outcomes, staff members recorded observations after each VR session regarding the participant’s response, including any incidents or concerns arising during or immediately after VR exposure. These observations are reported descriptively.

### 2.5 Data analysis

Descriptive statistics (means, standard deviations, frequencies, and percentages, as appropriate) were used to summarize participant characteristics, feasibility outcomes, and behavioral indicators.

Repeated continuous outcomes collected before and after each VR session (physiological measures and subjective VAS ratings) were analyzed using linear mixed-effects models. Separate models were fitted for the two units because they differed in their patient populations, staffing, and service-delivery conditions. Consequently, a between-unit effect would have been confounded with setting-level differences and could not be interpreted as an effect of participant characteristics. In each model, Moment (pre-session vs. post-session), Session (modeled as an eight-level categorical factor), and the Moment × Session interaction were specified as fixed effects. A random intercept for participant was included to account for the correlation between repeated observations from the same individual. This covariance structure was preferred over diagonal and first-order autoregressive alternatives on the basis of the Akaike Information Criterion. Models were estimated by restricted maximum likelihood, and denominator degrees of freedom were obtained using the Satterthwaite approximation. All available observations were used without imputation, under a missing-at-random assumption, and no covariates were included. Estimated marginal means, estimated pre-post changes with 95% confidence intervals (95% CI), and *p* values are reported. Changes in STAI-State scores between baseline and post-intervention were analyzed using paired-samples *t*-tests among participants with complete data at both time points (UHRPI, *n* = 21; UTJJ, *n* = 6).

Behavioral indicators were analyzed descriptively at participant level. For each indicator, means and standard deviations of the number of recorded events were calculated separately for each study phase and clinical setting. In addition, the number of participants contributing at least one recorded event was determined for each indicator, phase, and setting, in order to indicate the extent to which mean frequencies reflected events distributed across participants rather than concentrated in a small number of individuals. Given the exploratory nature of these outcomes, the small sample size, and the low frequency of several events with a high proportion of zero values, no inferential analyses were performed for behavioral indicators, and no comparisons between phases or settings were tested.

All analyses were performed using IBM SPSS Statistics version 26.0. Statistical significance was set at *p* < 0.05. No adjustment for multiple comparisons was applied. Because the clinical outcomes were secondary and exploratory rather than confirmatory, unadjusted *p* values are reported together with estimated changes and 95% confidence intervals.

### 2.6 Ethical approval and consent

Ethical approval was obtained from the Research Ethics Committee of Fundación Sant Joan de Déu (reference number PS-05-24). Capacity to provide informed consent was a predefined inclusion criterion, assessed for each candidate by the treating clinician during the eligibility review; no standardized capacity instrument was used. Four UHRPI participants had a diagnosis of mild intellectual disability; all had full legal capacity and consented on their own behalf, with the study information additionally explained verbally and the participant asked to describe the procedure in their own words before signing. Written informed consent was obtained from all adult participants. For participants under 18 years of age, written informed consent was obtained from the parent or legal guardian, and the participant also gave written consent in person, in accordance with Spanish legislation on biomedical research involving minors. No participant was subject to court-appointed support measures. Enrolment and consent were carried out by the research team rather than by the treating clinicians. Participation was voluntary, participants could withdraw at any time without giving a reason, and participation or non-participation did not affect access to, or the quality of, standard care.

## 3 Results

### 3.1 Feasibility: acceptability and usability of the intervention

Acceptability scores, reported by service users, were high in both groups (Table 2). Mean satisfaction was 8.67 (SD = 1.46) in the UHRPI and 8.00 (SD = 1.07) in the UTJJ. Comfort was rated 8.81 (SD = 1.25) and 8.25 (SD = 1.16), in the UHRPI and UTJJ, respectively. Willingness to continue using VR was 8.38 (SD = 2.01) in the UHRPI and 8.75 (SD = 1.91) in the UTJJ. Perceived usefulness for emotional management was 8.19 (SD = 1.86) in the UHRPI and 6.88 (SD = 2.42) in the UTJJ.

**Table 2:** Feasibility (acceptability and usability) of the VR-based relaxation intervention.

Variable	UHRPI (participants *n* = 21)		UTJJ (participants *n* = 8)	
	*M*	SD	*M*	SD
Service user acceptability				
Satisfaction	8.67	1.46	8.00	1.07
Comfort	8.81	1.25	8.25	1.16
Willingness to continue using VR	8.38	2.01	8.75	1.91
Usefulness for emotional management	8.19	1.86	6.88	2.42

Variable	UHRPI (assessments *n* = 21)		UTJJ (assessments *n* = 8)	
	*M*	SD	*M*	SD
Professional usability				
Willingness to use the device	5.00	0.00	5.00	0.00
Ease of use	5.00	0.00	5.00	0.00
Learnability	5.00	0.00	5.00	0.00
Need for assistance	1.76	1.61	1.00	0.00
Comfort	5.00	0.00	5.00	0.00
Perceived safety	5.00	0.00	5.00	0.00
Applicability in routine clinical practice	5.00	0.00	5.00	0.00

Values represent means (M) and standard deviations (SD). Service-user acceptability was assessed once after completion of the intervention using a 0–10 visual analog scale (VAS). Professional usability was assessed at the end of each participant’s four-week intervention phase using a 5-point Likert scale completed by a professional involved in delivering the VR intervention, yielding one assessment per participant (UHRPI, *n* = 21 assessments completed by 6 professionals; UTJJ, *n* = 8 assessments completed by 3 professionals). Higher scores indicate greater acceptability or usability for all positively worded items; for the “Need for assistance” item, higher scores indicate greater perceived need for assistance.

Professional usability was evaluated through 29 usability assessments, with one questionnaire completed at the end of each participant’s four-week VR intervention phase by a professional involved in delivery (UHRPI, *n* = 21 assessments completed by 6 professionals; UTJJ, *n* = 8 assessments completed by 3 professionals; response rate 100%). Usability ratings were high for the six positively worded items in both settings (Table 2), with maximum mean scores for willingness to use the device, ease of use, learnability, comfort, perceived safety, and applicability in routine clinical practice. The seventh item was worded in the opposite direction, so that higher scores indicate a greater perceived need for support; mean scores on this item were 1.76 (SD = 1.61) in the UHRPI and 1.00 (SD = 0.00) in the UTJJ, indicating that professionals in both settings reported little or no need for assistance in operating the device. Because responses to the remaining six items reached the maximum possible score in both settings, usability results are presented descriptively. Across both settings, staff identified some practical considerations for future implementation, including occasional playback, synchronization, and battery-life issues in the UHRPI and perceived excessive video duration in the UTJJ.

All 29 participants completed all eight scheduled VR sessions during the intervention phase, resulting in 232 of 232 planned sessions delivered, with no dropouts, no missed or canceled sessions, and no protocol deviations. Data completeness was high across outcomes. Physiological assessments were available at 333 of 336 planned pre- and post-session measurement points in the UHRPI (two pre-session and one post-session measurement at session 3 were not recorded, involving two participants) and at all 128 planned points in the UTJJ. Session-based VAS assessments were available at 3,233 of 3,248 planned measurement points; for one participant in the UHRPI the scales were not completed at one session, and one further item was missing at another session. For the STAI-State, post-intervention data were unavailable for two participants in the UTJJ; therefore, paired analyses of state anxiety were based on complete pre- and post-intervention data from 21 participants in the UHRPI and 6 participants in the UTJJ.

The intervention was generally well tolerated in both settings. Clinical staff recorded observations and any relevant incidents following each VR session. In the UHRPI, one episode of dizziness was recorded during a VR session, without reported clinical consequences or discontinuation of the intervention. In the UTJJ, no adverse physical effects related to VR use were recorded.

### 3.2 Clinical changes: physiological measures and state anxiety

Pre–post changes in physiological measures across the eight VR sessions were examined using linear mixed-effects models (Table 3). In the UHRPI, significant reductions were observed in systolic blood pressure [*F*(1, 297.00) = 27.425, *p* < 0.001] and heart rate [*F*(1, 297.01) = 9.623, *p* = 0.002]. Diastolic blood pressure also decreased, but this change did not reach statistical significance [*F*(1, 297.02) = 3.251, *p* = 0.072]. In the UTJJ, no significant pre–post changes were observed in systolic blood pressure [*F*(1, 105.00) = 3.858, *p* = 0.052], diastolic blood pressure [*F*(1, 105.00) = 0.030, *p* = 0.863], or heart rate [*F*(1, 105.00) = 1.140, *p* = 0.288].

**Table 3:** Pre-post changes in physiological measures and state anxiety during the VR-based relaxation intervention.

Unit	Outcome	Pre-intervention Mean (SD)	Post-intervention Mean (SD)	Estimated change (95% CI)	Statistic	*p*
UHRPI	Systolic BP	121.69 (11.90)	116.84 (11.10)	−4.79 (−6.59 to −2.99)	*F*(1, 297.00) = 27.425	<0.001***
	Diastolic BP	78.12 (8.82)	76.91 (8.50)	−1.18 (−2.47 to 0.11)	*F*(1, 297.02) = 3.251	0.072
	Heart rate	79.08 (14.84)	76.28 (14.88)	−2.76 (−4.52 to −1.01)	*F*(1, 297.01) = 9.623	0.002**
	STAI-state	37.76 (28.45)	23.76 (22.51)	−14.00 (−27.77 to −0.24)	t(20) = −2.12	0.047*
UTJJ	Systolic BP	123.16 (12.13)	120.58 (13.63)	−2.58 (−5.18 to 0.02)	*F*(1, 105.00) = 3.858	0.052
	Diastolic BP	69.97 (7.71)	70.16 (10.46)	0.19 (−1.96 to 2.33)	F(1, 105.00) = 0.030	0.863
	Heart rate	80.67 (16.83)	78.97 (16.36)	−1.70 (−4.87 to 1.46)	F(1, 105.00) = 1.140	0.288
	STAI-state	59.17 (19.40)	52.67 (19.42)	−6.50 (−37.75 to 24.75)	t(5) = − 0.54	0.616

Pre- and Post-Intervention values are presented as Mean (SD). For blood pressure and heart rate, values represent measurements taken before and after each of the eight VR sessions; these outcomes were analyzed using linear mixed-effects models fitted separately for each unit, with Moment (pre-session vs. post-session), Session and the Session × Moment interaction as fixed effects and a random intercept for participant. Models were based on 333 observations from 21 participants in the UHRPI and 128 observations from 8 participants in the UTJJ. Estimated changes represent model-estimated differences from pre-session to post-session, with 95% confidence intervals (CI); negative values indicate a decrease. State anxiety (STAI-State) was assessed at baseline and post-intervention only and was analyzed using paired-samples *t*-tests among participants with complete data at both time points (UHRPI, *n* = 21; UTJJ, *n* = 6); values are percentile scores. *p < 0.05, **p < 0.01, ***p < 0.001.

State anxiety (STAI-State), assessed at baseline and post-intervention, decreased significantly in the UHRPI (mean change = −14.00, 95% CI [−27.77, −0.24], *t*(20) = −2.12, *p* = 0.047), whereas no significant change was observed in the UTJJ (mean change = −6.50, 95% CI [−37.75, 24.75], *t*(5) = −0.54, *p* = 0.616).

### 3.3 Behavioral indicators across study phases

Behavioral indicators derived from standardized institutional records are summarized descriptively in Table 4.

**Table 4:** Participant-level behavioral indicators across the three study phases.

UHRPI (*n* = 21)						
Indicator	PRE Mean (SD)	*n*	VR Mean (SD)	*n*	POST Mean (SD)	*n*
Behavioral incidents requiring professional intervention	0.24 (0.89)	2	0.14 (0.48)	2	0.10 (0.30)	2
Violence toward peers	0.38 (0.67)	6	0.52 (1.17)	6	0.43 (0.60)	8
Violence toward staff	0.24 (0.63)	3	0.38 (0.81)	5	0.19 (0.68)	2
Violence involving center property	0.10 (0.30)	2	0.10 (0.30)	2	0.05 (0.22)	1
Substance use	0.00 (0.00)	0	0.05 (0.22)	1	0.05 (0.22)	1
Separation from environment	0.19 (0.51)	3	0.14 (0.36)	3	0.00 (0.00)	0
Observation	0.05 (0.22)	1	0.14 (0.36)	3	0.10 (0.30)	2
Mechanical restraint	0.00 (0.00)	0	0.00 (0.00)	0	0.05 (0.22)	1
Request for extra medication	4.29 (5.56)	14	3.48 (5.06)	14	3.33 (4.76)	13
Request for help due to anxiety	3.19 (3.44)	14	2.81 (3.16)	15	1.95 (2.56)	13

UTJJ (*n* = 8)						
Indicator	PRE Mean (SD)	*n*	VR Mean (SD)	*n*	POST Mean (SD)	*n*
Behavioral incidents requiring professional intervention	2.13 (2.48)	5	3.88 (4.29)	7	1.38 (1.77)	5
Violence toward peers	2.63 (2.00)	6	0.38 (1.06)	1	1.13 (0.99)	6
Violence toward staff	2.50 (2.00)	8	0.38 (0.74)	2	0.38 (0.52)	3
Violence involving center property	0.50 (1.07)	2	1.38 (1.69)	5	0.38 (0.52)	3
Substance use	0.13 (0.35)	1	0.00 (0.00)	0	0.13 (0.35)	1
Separation from environment	2.63 (1.60)	7	1.00 (1.93)	2	0.75 (1.04)	4
Observation	0.50 (0.76)	3	0.13 (0.35)	1	0.00 (0.00)	0
Mechanical restraint	0.13 (0.35)	1	0.00 (0.00)	0	0.00 (0.00)	0
Request for extra medication	1.50 (2.67)	5	1.25 (2.05)	4	0.63 (1.19)	2
Request for help due to anxiety	0.88 (1.13)	4	3.25 (3.24)	5	0.13 (0.35)	1

Values are means (M) and standard deviations (SD) of the number of recorded events per participant during each consecutive four-week study phase. PRE = pre-intervention; VR = intervention phase; POST = post-intervention follow-up. *n* denotes the number of participants who contributed at least one recorded event in that phase (UHRPI, out of 21; UTJJ, out of 8). Indicators were extracted from each participant’s own institutional clinical record using a standardized extraction form and therefore refer to study participants only.

In the UHRPI, mean frequencies of request for help due to anxiety and request for extra medication were lower during the VR phase than during the pre-intervention phase and remained lower during the post-intervention phase. Mean frequencies of behavioral incidents requiring professional intervention and separation from environment also decreased across the study phases. Small increases during the VR phase were observed for violence toward peers, violence toward staff, observation, and substance use, although mean frequencies remained low throughout the study. With the exception of requests for extra medication and requests for help due to anxiety, which involved 13 to 15 of the 21 participants in each phase, all indicators in the UHRPI were recorded for eight or fewer participants per phase.

In the UTJJ, mean frequencies of separation from environment, observation, mechanical restraint, and request for extra medication were lower during the VR and post-intervention phases than during the pre-intervention phase. In contrast, behavioral incidents requiring professional intervention, violence involving center property, and request for help due to anxiety showed higher mean frequencies during the VR phase, followed by lower values during the post-intervention phase. Violence toward peers and violence toward staff showed the highest mean frequencies during the pre-intervention phase and lower mean frequencies during the VR and post-intervention phases. All eight participants had at least one recorded incident of violence toward staff during the pre-intervention phase, compared with two participants during the VR phase and three during the post-intervention phase.

### 3.4 Subjective appraisals

Subjective appraisals measured immediately before and after each VR session were analyzed using linear mixed-effects models (Table 5).

**Table 5:** Pre–post changes in subjective emotional states.

UHRPI							
VAS dimension	PRE Mean (SD)	POST Mean (SD)	Estimated change (95% CI)	*F*(1, ~298) moment	*p*	*F*(7, ~298) Session × moment	*p*
Stress	3.00 (2.96)	1.55 (2.31)	−1.45 (−1.91 to −0.99)	38.172	<0.001***	0.229	0.978
Relaxation	6.25 (3.01)	7.56 (2.75)	+1.31 (0.82 to 1.80)	27.965	<0.001***	0.536	0.807
Anxiety	3.23 (3.19)	1.93 (2.38)	−1.30 (−1.76 to −0.84)	31.058	<0.001***	0.433	0.881
Sadness	2.52 (3.05)	2.03 (2.83)	−0.50 (−0.96 to −0.04)	4.481	0.035*	0.661	0.705
Happiness	6.16 (2.97)	6.71 (2.69)	+0.56 (0.13 to 0.99)	6.422	0.012*	0.532	0.810
Connection with nature	3.88 (3.72)	7.10 (3.25)	+3.22 (2.73 to 3.72)	164.539	<0.001***	0.406	0.899
Craving	2.09 (2.94)	1.75 (2.71)	−0.33 (−0.82 to 0.16)	1.797	0.181	1.165	0.323

UTJJ							
VAS dimension	PRE Mean (SD)	POST Mean (SD)	Estimated change (95% CI)	*F*(1, 105) moment	*p*	*F*(7, 105) Session × moment	*p*
Stress	4.09 (2.34)	2.61 (2.29)	−1.48 (−2.21 to −0.76)	16.465	<0.001***	0.923	0.492
Relaxation	3.86 (2.44)	5.73 (2.72)	+1.88 (1.08 to 2.68)	21.619	<0.001***	0.206	0.983
Anxiety	5.03 (2.64)	3.45 (2.23)	−1.58 (−2.39 to −0.76)	14.692	<0.001***	0.428	0.883
Sadness	3.08 (2.38)	2.39 (1.84)	−0.69 (−1.32 to −0.05)	4.590	0.034*	0.320	0.944
Happiness	4.25 (2.61)	4.73 (2.73)	+0.48 (−0.27 to 1.24)	1.617	0.206	0.144	0.994
Connection with nature	2.06 (2.28)	5.75 (2.82)	+3.69 (2.82 to 4.55)	71.485	<0.001***	0.522	0.816
Craving	2.59 (2.25)	1.95 (2.21)	−0.64 (−1.24 to −0.04)	4.466	0.037*	1.059	0.395

Values are presented as mean (SD). Visual analog scale (VAS) ratings (range 0–10) were obtained immediately before and after each of the eight VR sessions and were analyzed using linear mixed-effects models fitted separately for each unit, with Moment (pre-session vs. post-session), Session (included to account for variation across sessions; main effect not reported) and the Session × Moment interaction as fixed effects and a random intercept for participant. Models were estimated by restricted maximum likelihood, and denominator degrees of freedom were obtained by Satterthwaite approximation. Denominator degrees of freedom varied marginally between 297.0 and 298.1 in the UHRPI models and were 105 in all UTJJ models; column headings give the approximate value. Models were based on 334 observations from 21 participants in the UHRPI (333 for Sadness) and 128 observations from 8 participants in the UTJJ. Estimated changes represent model-estimated differences from pre-session to post-session, with 95% confidence intervals (CI); negative values indicate a decrease. A significant effect of Moment indicates an overall pre–post change across sessions, whereas a significant Session × Moment interaction would indicate that the magnitude of the pre–post change differed between sessions. *p < 0.05, **p < 0.01, ***p < 0.001.

In the UHRPI, significant main effects of Moment were observed for Stress, Relaxation, Anxiety, Sadness, Happiness, and Connection with nature (all *p* < 0.05), whereas the change in Craving did not reach statistical significance (*p* = 0.181). In the UTJJ, significant main effects of Moment were observed for Stress, Relaxation, Anxiety, Sadness, Connection with nature, and Craving (all *p* < 0.05), whereas the change in Happiness did not reach statistical significance (*p* = 0.212).

No significant Session × Moment interactions were observed for any subjective appraisal measure in either unit, suggesting that the magnitude of the immediate pre–post changes did not differ systematically across the eight VR sessions.

## 4 Discussion

### 4.1 Feasibility, acceptability, and usability

The present feasibility study indicates that an immersive VR-based relaxation intervention can be implemented within routine care in forensic mental healthcare. Service users in both units reported high satisfaction, comfort, and willingness to continue using VR, and professionals rated willingness to use the device, learnability, comfort, perceived safety, and applicability in routine practice favorably. Users’ high satisfaction scores and their willingness to continue using VR indicate that immersive relaxation programs are experienced as acceptable rather than intrusive or aversive, even in highly structured, risk-focused environments. These findings add to evidence that VR-based interventions are acceptable and usable in mental healthcare settings (Ilioudi et al., 2023; Riches et al., 2023), and extend this to forensic contexts, where emerging research has likewise reported feasibility and promising clinical utility (Sygel and Wallinius, 2021; Woicik et al., 2023; Mertens and Van Gelder, 2025).

The fact that the intervention was delivered by regular unit staff after brief training, with limited preparation time and a small number of technical issues, suggests that VR can be operated with minimal disruption to routine workflows (Woicik et al., 2023). The low preparation demands and limited staff involvement further support its integration into secure services, where time and resources are constrained (Andersson et al., 2026). Occasional playback and synchronization problems, battery-life limitations, and perceived excessive video duration indicate that remaining barriers are mainly technical and procedural and could be addressed through minor refinements and clear local procedures.

### 4.2 Clinical changes: physiological and subjective state

The pre–post analyses of physiological and subjective measures suggest that VR-based relaxation was associated with short-term reductions in physiological arousal and anxiety, particularly in the adult unit, whereas changes in the juvenile unit were more variable and did not reach statistical significance. In the adult unit, significant reductions in systolic blood pressure, heart rate, and state anxiety suggest that brief VR sessions can produce measurable short-term decreases in autonomic arousal, in line with previous work showing that immersive VR interventions can reduce anxiety- and stress-related symptoms in clinical populations (Holopainen et al., 2023). Diastolic blood pressure also decreased, although this change did not reach statistical significance. In the juvenile unit, changes in physiological indices and anxiety did not reach significance, which may reflect limited statistical power and greater variability in this smaller subgroup; for STAI-State, complete pre-post data were available for only six participants.

Across both units, service users reported significant reductions in stress and anxiety and significant increases in relaxation and connection with nature supporting the interpretation that immersive naturalistic environments can provide relief from distress and negative affect (Hartig et al., 2014; White et al., 2019), even when simulated or in virtual format (Kahn et al., 2009; Calogiuri et al., 2018; Browning et al., 2020). No significant Session × Moment interactions were observed for any subjective appraisal measure in either setting, suggesting that the magnitude of these immediate pre-post changes did not differ systematically across the different VR sessions. From a clinical and risk-management perspective, such changes are relevant because aggressive behavior in forensic settings is often preceded by elevations in arousal and other early signs of escalation (de Looff et al., 2019). Our findings align with earlier studies suggesting that VR-based interventions can contribute to reductions in aggression, improvements in emotion regulation, and more adaptive observable behavior, even when effects on broader clinical or physiological outcomes are less consistent (Woicik et al., 2023; González Moraga et al., 2024). In this context, immersive relaxation approaches may facilitate rapid reductions in distress and help individuals in regaining a sense of control over their emotional state (Veling et al., 2021; Klein Haneveld et al., 2023).

### 4.3 Behavioral indicators across study phases

Although changes in behavioral indicators across the pre-, intervention, and post-intervention phases were generally small and mixed, they may still be clinically informative. In the adult unit, mean frequencies of requests for anxiety-related support and additional medication decreased during and after the intervention period, accompanied by modest reductions in certain restrictive measures involving separation from the environment. In the juvenile unit, requests for additional medication, the use of restrictive measures, and violent incidents toward peers and staff showed lower mean frequencies during and after the intervention period than before it, although some behaviors (e.g., anxiety-related help-seeking, behavioral incidents requiring staff intervention, and violent incidents involving property) showed transient increases during the intervention phase before declining again in the post-intervention period.

In high-risk forensic environments, even such modest patterns are relevant. Reductions in restrictive practices and requests for additional medication align with broader efforts to reduce coercive practices and promote recovery-oriented and trauma-informed care (Huckshorn, 2004; LeBel et al., 2010; Marzano et al., 2016; Jalil et al., 2017; Kramarz et al., 2023). Interventions that support timely de-escalation may contribute not only to individual well-being but also to safer ward environments and improved therapeutic climates. They illustrate how VR-based relaxation might potentially translate into everyday clinical practice, as even modest reductions in anxiety-related help-seeking, *ad hoc* medication requests, or restrictive interventions could, over time, alleviate staff burden and contribute to a more stable therapeutic climate. The largest observed differences occurred between the pre-intervention phase and the subsequent phases in the juvenile unit, where baseline event frequencies were highest. Given the descriptive nature of these indicators, the small sample and the absence of a control group, such patterns are also compatible with regression to the mean, changes in acuity, or variation in recording practice, and cannot be attributed to the intervention.

### 4.4 Clinical and organizational implications

From a clinical perspective, the present findings suggest that VR-based relaxation may be useful as a brief tool to support emotion regulation, either as routine care or during periods of heightened distress. The observed reductions in stress and anxiety, alongside increases in relaxation and descriptive improvements in some behavioral indicators, indicate that immersive environments can facilitate rapid shifts in affective state. VR-assisted relaxation may therefore complement existing de-escalation strategies by providing a low-threshold option that can be deployed before more intensive or restrictive measures are required.

In forensic settings, tailoring VR scenarios to individual risk and need profiles aligns with proposals to integrate such interventions within the risk–need–responsivity framework (Kip and Bouman, 2021). VR should thus be applied as a flexible, targeted tool by adapting immersive scenarios to service users’ specific triggers and treatment needs, thereby enhancing clinical relevance and engagement (Mertens and Van Gelder, 2025; Sivermo et al., 2025). In the present study, improvements in perceived connection with nature were accompanied by reductions in stress and anxiety, consistent with evidence on the restorative effects of natural environments on emotional and physiological stress (Hartig et al., 2014; White et al., 2019), including when such environments are delivered through simulated or virtual formats (Kahn et al., 2009; Calogiuri et al., 2018; Browning et al., 2020). Similarly, biofeedback-driven applications illustrate how real-time physiological signals can be used to support breathing regulation and reduce anxiety and hostility (Bossenbroek et al., 2020; Weerdmeester et al., 2021; Klein Haneveld et al., 2023, 2025). Building on these approaches, VR enables the adaptation of virtual scenarios to elicit clinically relevant emotional and behavioral responses (Woicik et al., 2023; González Moraga et al., 2024). It offers features such as personalization and real-time interaction, allowing scenarios to be aligned with specific treatment needs, including the simulation of real-world situations and environments that are inaccessible due to forensic restrictions or unsafe to reproduce in practice. Through its immersive and experiential nature, VR allows service users to practice adaptive responses in a safe and controlled environment, which may support the acquisition and generalization of more functional emotion regulation strategies and adaptive behaviors (Van Wolffelaar et al., 2024).

From an organizational perspective, the intervention functioned as a low-burden strategy: sessions were brief, repeatable, and required limited preparation, while being deliverable by regular unit staff after short training. In forensic services with high workload, security demands, and constrained resources, VR-based approaches may therefore serve as a rapid and readily deployable adjunct to existing strategies for emotion regulation and other identified treatment needs. At the same time, staff reports of technical issues and content-related considerations underscore that successful implementation depends on more than clinical promise alone. Consistent with broader work on eHealth technology in forensic mental healthcare, our findings underline that usability, organizational readiness, and clear implementation strategies are critical to realizing the benefits of VR (Kip et al., 2018; Andersson et al., 2026). That implementation context shapes outcomes is illustrated by a recent multi-site evaluation of a VR-enhanced training program in European residential child and adolescent mental health care, in which improvements in trauma-informed attitudes differed markedly between participating sites (Kostadinov et al., 2026). Usability, organizational readiness, and clear implementation strategies are therefore key prerequisites for sustainable use and point to the need for targeted technical refinements, careful selection and tailoring of VR content to clinical needs, clear local protocols, and wider staff training to support safe, consistent, and long-term integration into routine care.

### 4.5 Strengths and limitations

This study has several strengths. The intervention was implemented within routine clinical practice, providing ecologically valid insights into feasibility and real-world application in secure settings. The inclusion of both adult and youth populations allowed examination across distinct forensic mental healthcare settings and populations. In addition, the use of multiple outcome domains, including physiological, behavioral, and subjective, offers a comparatively comprehensive assessment of the intervention’s preliminary outcomes. Finally, delivery by regular unit staff after brief training supports its potential for integration into standard care pathways.

Several limitations should nevertheless be considered. Participants were identified by the clinical teams of each unit rather than through consecutive or systematic screening, and the numbers screened, considered eligible, invited or declining were not recorded. Selection bias can therefore not be excluded, and its extent cannot be quantified. This may have contributed to the high acceptability observed and limits the generalizability of these findings, particularly since acceptability was a primary outcome. Six of the seven usability items reached the maximum score with no variance in both settings, so the usability findings indicate the absence of major usability barriers rather than a graded assessment and undifferentiated responding cannot be excluded. Sample sizes were modest, particularly in the juvenile unit, which constrained statistical power and the precision of subgroup analyses. The UHRPI is a male-only unit, so the findings from the adult setting apply to men only. The absence of a control or comparison group limits causal inference and makes it difficult to disentangle intervention effects from concurrent clinical changes. Diagnostic heterogeneity likely introduced additional variance, potentially obscuring differences in response. The follow-up period was short, precluding firm conclusions about the durability of effects, possible habituation, or longer-term behavioral change. Moreover, outcomes relied partly on self-report and routine clinical documentation, which may be affected by reporting biases and variability in recording practices over time.

The small number of participants, particularly in the juvenile unit, further limited the precision of the mixed-effects estimates. This was compounded by missing post-intervention STAI-State data for two UTJJ participants, resulting in only six complete pre-post observations for this outcome. In addition, STAI-State was used as percentile scores, which are bounded and not normally distributed; the paired comparisons for this outcome should therefore be interpreted with caution. Because the clinical outcomes were secondary and exploratory and no adjustment for multiple comparisons was applied, nominally significant results should be interpreted as provisional and require confirmation in adequately powered controlled studies. Behavioral indicators were analyzed descriptively and should therefore not be interpreted as evidence of intervention effects. They were mostly low-frequency events recorded for a small number of participants, so that phase differences in mean frequencies may reflect the behavior of individual participants rather than group-level patterns, and they depend on what unit staff observed and entered in the clinical record. Since staff were aware of which phase a participant was in, an influence of the intervention on recording behavior can therefore not be excluded. Information on medication changes during the study, previous VR experience, concurrent treatment, and standardized illness severity was not systematically collected, which limits the interpretation of both the clinical and the behavioral findings.

Future research should therefore prioritize controlled study designs with larger samples to evaluate the effectiveness of VR-based interventions in forensic populations. Further work is needed to determine whether responses differ across age groups and settings, using designs that can separate the two. Further work is also needed to identify which types of VR content are best suited to specific clinical targets. In addition, examining whether session-level improvements translate into sustained behavioral and clinical changes will be essential for determining long-term impact. To address these questions, combining physiological, behavioral, and subjective measures will be important for achieving a comprehensive understanding of intervention effects.

## 5 Conclusion

This feasibility study indicates that immersive VR-based relaxation can be implemented within routine forensic mental healthcare and is experienced as acceptable and usable by both service users and staff. Beyond feasibility, the findings suggest that brief VR sessions may support reductions in subjective and physiological indicators of stress and anxiety, alongside increases in perceived relaxation and connection with nature. Additional reductions in physiological indicators of arousal were observed in the UHRPI. Although behavioral indicators showed only descriptive and mixed changes, the patterns observed across study phases may warrant further investigation of their relevance to everyday clinical practice in forensic settings. These results suggest that VR-based relaxation may warrant evaluation as a brief, complementary tool for emotion regulation and early de-escalation in secure settings, particularly where access to calming environments such as nature is constrained. De-escalation was not among the outcomes assessed here, and evaluating this possibility would require studies in which it is specified as an outcome. Given the exploratory design, small sample sizes, and absence of a control condition, these findings should be interpreted cautiously. Future research with larger samples, longer follow-up, and systematic implementation evaluations is needed to determine effectiveness, safety, mechanisms of change, and conditions for sustainable integration into routine forensic care.

## Data Availability

The de-identified data that support the findings of this study are available from the corresponding author upon reasonable request. Because the sample consists of service users from two forensic mental health services, including minors, and the data include information extracted from institutional clinical records, combinations of demographic and clinical variables may allow re-identification; data sharing is therefore subject to ethical and legal restrictions and will require a data sharing agreement.
